# Tissue-Specific Modulation of Gluco- and Growth-Regulatory Factor Abundance by Nesfatin-1 and Nesfatin-1-like Peptide in Goldfish

**DOI:** 10.3390/ani13091437

**Published:** 2023-04-22

**Authors:** Jithine Jayakumar Rajeswari, Suraj Unniappan

**Affiliations:** 1 Department of Veterinary Biomedical Sciences, Western College of Veterinary Medicine, University of Saskatchewan, Saskatoon, SK S7N 5B4, Canada; 2 Department of Biological Sciences, University of Calgary, 507 Campus Dr NW, Calgary, AB T2N 4V8, Canada

**Keywords:** nesfatin-1, nesfatin-1-like peptide, muscle, adipose, liver, glucose transporters, insulin-like growth factor

## Abstract

**Simple Summary:**

Nesfatin-1 and nesfatin-1-like peptide (Nlp) have hormone-like biological actions in vertebrates. Their effects on glucose homeostasis and growth hormone are well documented in mammals, but information on this topic is limited in fish. We tested if nesfatin-1 and Nlp regulate factors that are key in glucose homeostasis and growth in goldfish using both in vivo and in vitro approaches. The results of in vivo and in vitro studies suggest that both nesfatin-1 and Nlp are insulinotropic (adipose tissue), promote glucose uptake (adipose tissue and muscle), and regulate the Gh-Igf axis (liver) in goldfish.

**Abstract:**

Nesfatin-1 and nesfatin-1-like peptide (Nlp) are derived from precursors nucleobindin-2 and -1, two calcium and DNA binding proteins, respectively. Both peptides exhibit hormone-like actions in mammals and fish. These functions include insulinotropic effects of nesfatin-1 and Nlp seen in mice and their growth hormone suppressive actions reported in goldfish. We hypothesized that nesfatin-1 and Nlp are insulin stimulatory (in adipose tissue) and modulate growth hormone and insulin-like growth factors and glucose transporters in goldfish. To test this, goldfish were intraperitoneally injected with either nesfatin-1 or Nlp (50 ng/g BW) or saline alone (control) and sampled at one-hour post-injection (in vivo study). In a separate study, tissue samples were collected and were incubated with either nesfatin-1 or Nlp for one or six hours (in vitro study). Transcript (mRNA) abundance data from the adipose tissue suggest that both nesfatin-1 and Nlp significantly upregulate the abundance of preproinsulin, insulin receptors, and *pcsk1* and *pcsk2* mRNAs. Meanwhile, the abundance of preproglucagon mRNA in the adipose tissue was significantly downregulated in both in vivo and in vitro studies. These results agree with the insulinotropic and glucagonostatic roles for nesfatin-1 and Nlp reported in rodents. The transcript abundance of growth regulators (*igf1*, *igf2a*, and *ghra*) and glucose transporters (*slc2a2* and *slc5a1*) were upregulated in the muscle, while an opposite effect on these mRNAs was found in the liver of goldfish following nesfatin-1 and Nlp administration. Our results suggest that both nesfatin-1 and Nlp have tissue-specific regulatory roles on growth and glucoregulatory elements in the liver and muscle of goldfish. This agrees with our previous studies that showed a suppressive action of nesfatin-1 on growth hormone in goldfish liver. The results presented here provide strong supportive/confirmatory evidence for tissue-specific insulinotropic and gluco- and growth-regulatory actions of nesfatin-1 and Nlp in goldfish.

## 1. Introduction

Nesfatin-1 is an 82 amino acid peptide processed by prohormone convertases from nucleobindin-2 (NUCB2). Over the last decade, significant information on the biological actions of nesfatin-1 has been reported in vertebrates, including fish. The main physiological roles of nesfatin-1 in fish include regulation of food intake [1,2], pituitary and gonadal hormone synthesis and/or secretion [3,4,5], cardiovascular functions [6], and oocyte maturation [5]. Research using zebrafish in our lab identified a nesfatin-1-like peptide (Nlp), which is processed from a precursor nucleobindin-1 (NUCB1) that has very high amino acid sequence similarity with NUCB2 [7]. Nlp shares a number of biological actions with nesfatin-1, mainly the regulation of food intake and hormone secretion [7,8]. Together, nesfatin-1 and Nlp are now considered to play key roles in the physiological homeostasis of fish.

Nesfatin-1 and/or Nlp regulation of hormone secretion from the pituitary gland and adrenal gland and its regulators from the brain and gonads are well characterized in goldfish, a model teleost in comparative endocrinology. Intraperitoneal injection of nesfatin-1 suppresses growth hormone (Gh) transcript abundance and Gh secretion in goldfish [3]. Nesfatin-1 exhibited the same inhibitory role on Gh secretion in vitro from goldfish pituitary cells. Gh stimulatory GnRH mRNA was also found decreased in the goldfish brain. In the same study, in vitro incubation of liver fragments with nesfatin-1 decreased insulin-like growth factor (*igf*) mRNA [3]. These results suggest a general suppressive effect for nesfatin-1 on Gh-Igf axis in goldfish. Meanwhile, nesfatin-1 stimulated both pituitary adrenocorticotrophic hormone (Acth) and cortisol in vivo in goldfish [9]. The suppressive role of nesfatin-1 on serum luteinizing hormone (Lh) and Lh beta and follicle-stimulating hormone (Fsh) beta mRNA was also reported in goldfish [5]. All these point to a major regulatory role for nesfatin-1 on pituitary and adrenal hormones. The effects appear to be cell-specific within the anterior pituitary gland.

The physiology of growth is dependent on feeding and metabolism. Nesfatin-1 is a suppressor of feeding (anorexigen) in fish [1,2]. Nlp also exerts a similar anorexigenic effect in fish [7]. In addition, both peptides exert comparable modulatory effects on appetite regulatory hormone mRNAs. Thus, nesfatin-1 and Nlp might negatively influence growth by restricting nutrient availability. It is also evident that nesfatin-1 directly modulates Gh, Igf, and other Gh-regulatory hormones, which might affect growth [3]. While suppressive effects in pituitary Gh and liver Igf for nesfatin-1 were reported previously, whether Nlp exhibits such effects is still unclear. Our research found that goldfish adipose tissue is a major source of insulin [10]. The goal of this research was to identify whether nesfatin-1 affects growth and glucoregulatory genes in specific metabolic tissues in goldfish and to determine whether Nlp has comparable effects in goldfish. We hypothesized that nesfatin-1 and Nlp have suppressive roles on both growth and glucoregulatory genes in goldfish metabolic tissues, namely, muscle, liver, and adipose tissue.

## 2. Materials and Methods

### 2.1. Animal and Ethics

Male and female adult (~2 years old) goldfish (*Carassius auratus*) of the common variety were purchased through the institutional animal order desk from a local vendor. Fish were housed in the animal care facility at the University of Saskatchewan in 300 L aquaria holding tanks. Dechlorinated, filtered (continuous flow) fresh water at 22 ± 2 °C with continuous aeration was used for housing the fish. Fish were maintained in a 12 h light:12 h dark (12L:12D) photoperiod (lights on at 07.00 h in the morning). Fish were fed once every morning at 10.00 h with a commercial pellet diet (Aqueon goldfish granules, Franklin, WI, USA). Fish were acclimated for 3 months in the conditions described above before commencing experiments. All experiments involving goldfish were completed from September–October when the goldfish were in the early recrudescence stage of their reproductive cycle [11].

### 2.2. Peptides and Reagents

Goldfish nesfatin-1 and Nlp were custom synthesized as described earlier [4,5,8]. Goldfish nesfatin-1 (1–82) [gfnesfatin-1; VPISIDKTKVKLPEETVKESPQNVDTGLHYDRYLREVIDFLEKDQHFREKLHNTDMEDIKQGKLAKELDFVSHHVRTKLDEL) (Genscript, NJ, USA) and the goldfish/zebrafish Nlp (1-77) [gf/zfNlp; VPIDRNPDPPQEEKAEENVDTGLYYDRYLREVIEVLETDPHFREKLQTANTEDIKNGRLSKELDLVGHHVRTRLDEL) (Pacific Immunology, Ramona, CA, USA) were synthesized and their purity (>95%) was confirmed by mass spectrometry and MALDI-TOF as described earlier [12]. For anesthesia (in vivo injection) and euthanasia (for tissue collection for both in vivo and in vitro studies), tricaine methanesulfonate-222 was used (TMS, 0.5% for few minutes for anesthesia and long-time exposure to 0.5% TMS following spinal transection for euthanasia, Syndel Laboratories, Qualicum Beach, BC, Canada).

### 2.3. In Vivo Studies

Two weeks before commencing the experiments, male and female goldfish (6 weight-matched fish per group, 22 ± 2 g) were randomly housed in 10 L aquaria (2 fish per aquaria, a total of 3 tanks with 6 fish in each tank for 1 control and 2 treatment groups) for acclimation. The water parameters and circadian cycle were the same as described above. Both nesfatin-1 and Nlp doses and time points for sampling were selected based on pilot studies and previous research [4,5,8]. Briefly, 24 h-fasted fish were anesthetized in TMS and were injected intraperitoneally (IP) with 100 μL of sterile saline (0.9% NaCl) containing (50 ng/g body weight) gfnesfatin-1 or gf/zfNlp. Control groups were injected with saline (100 μL with no peptides). At 1 h post-injection, control and treated fish (both nesfatin-1 and Nlp injected groups, 6 fish each) were euthanized as described above and adipose tissue (mesenteric) (as described by [10]), liver, and muscle were collected and flash frozen in liquid nitrogen and were stored at −80 °C until further analysis.

### 2.4. In Vitro Studies

The in vitro tissue culture study followed methods described earlier [10,13]. On the day of the experiment, adipose (mesenteric), liver, and muscle tissues were collected from 24 h-fasted goldfish. Immediately, tissue samples were placed on a sterile 24-well plate containing 1 mL of DMEM (Dulbecco’s modified Eagle medium; Thermo Fisher Scientific, Waltham, MA, USA) supplemented with 44 mM sodium bicarbonate, 1% penicillin-streptomycin, and 0.05% gentamicin (the modified media were called as DMEM-Plus). DMEM-Plus media (preincubation media) and the tissue samples were stabilized for 2 h at 23 °C in a cell culture incubator under an atmosphere of 5% CO2 and 95% O2. Following the pre-incubation, DMEM-Plus media was replaced with either DMEM-Plus media and nuclease-free water (for control) or DMEM-Plus media with either gf nesfatin-1 or gf/zfNlp (1 nmol/L or 10 nmol/L). The incubation periods used were 1 h (1 nmol/L) or 6 h (10 nmol/L). The dose and time points were selected based on pilot studies and previous research by our group [3]. Following incubation, the tissue samples were collected and immediately flash frozen in liquid nitrogen and stored at −80 °C until further analysis.

### 2.5. Total RNA Extraction, cDNA Synthesis, and qPCR

Total RNA extraction and cDNA synthesis were performed as previously described [4]. Briefly, total RNA from both in vitro and in vivo experimental samples were extracted using RiboZol RNA isolation reagent (aMReSCO, VWR, Canada) following manufacturer’s instructions. The quantity of total RNA samples was determined using a NanoDrop 2000 spectrophotometer (Thermo Fisher Scientific, Waltham, MA, USA). One microgram of total RNA from each sample was reverse transcribed to cDNA using iScript cDNA synthesis kit (Bio-Rad, Hercules, CA, USA). Real-time qPCR was performed using a CFX Connect Real-Time PCR Detection System (Bio-Rad, Hercules, CA, USA) and Universal SYBR Green Master Mix (Bio-Rad, Hercules, CA, USA). Primer details and the primer-specific annealing temperature are provided in Table 1. qPCR cycle conditions were initial denaturation/polymerase activation at 95 °C (3 min); 35 cycles of denaturation at 95 °C (10 s); specific annealing temperature (Table 1) (30 s); and a melting curve analysis (to confirm single amplicon) at 65 °C to 95 °C (5 s). Both 18S rRNA and β-actin were used as housekeeping genes for data normalization and the Livak method [14] was used for qPCR data analysis.

### 2.6. Statistical Analysis

One-way analysis of variance (one-way ANOVA) following Dunnett’s multiple comparisons test (compare between control and treatments, for in vivo study) or Tukey’s multiple comparisons test (for comparison between multiple groups, for in vitro studies) were used for statistical analysis. Prior to statistical tests, data were checked for normality and homogeneity of variance (Brown–Forsythe test) assumptions. Data that failed these assumptions were log-transformed and rechecked. Raw data (non-transformed) were used for generating graphs. *p* < 0.05 was considered statistically significant. Prism 8 (GraphPad, Boston, MA, USA) was used for generating graphs and statistical analysis. Data are presented as mean + SEM.

## 3. Results

### 3.1. Nesfatin-1 and Nlp Stimulated Insulin mRNA Abundance in Goldfish Adipose Tissue

**Figure 1:**
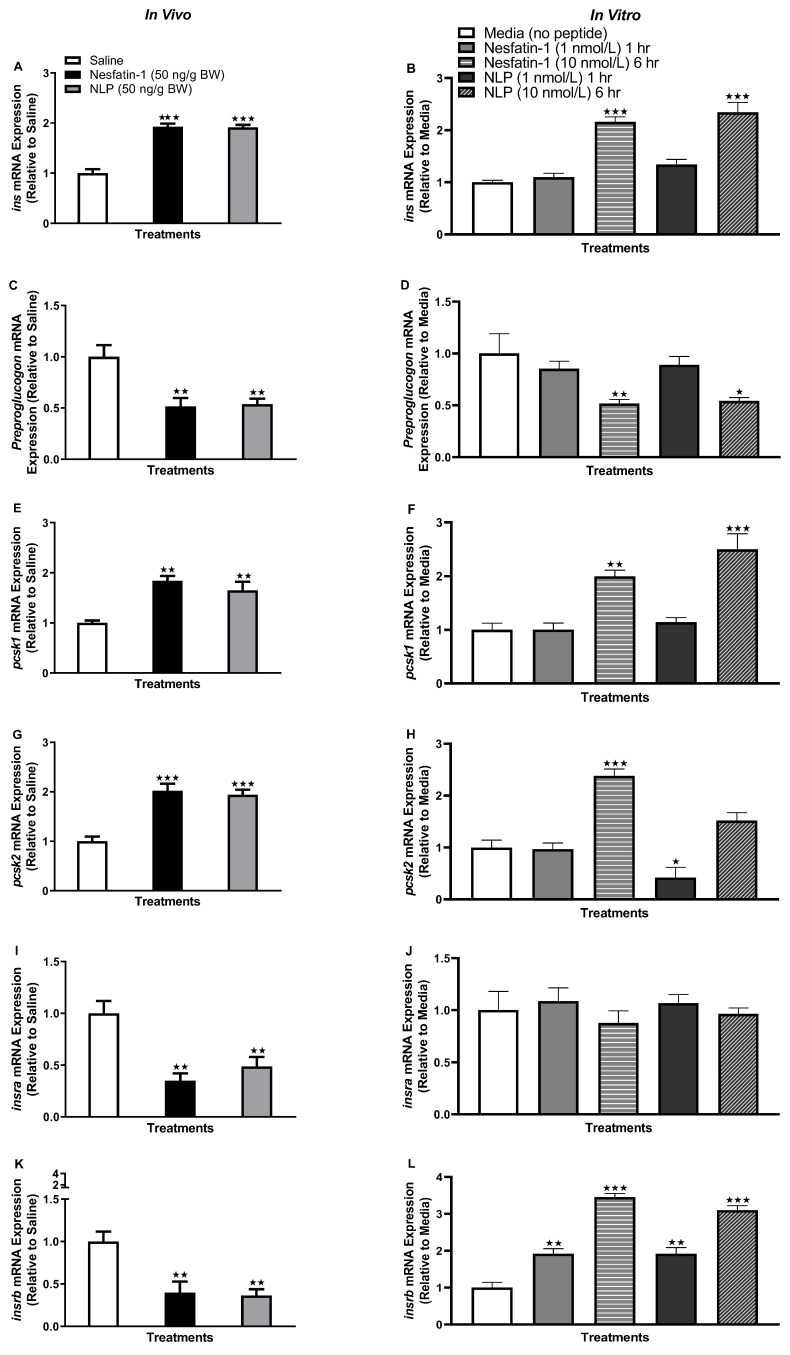
Effects of in vivo (left panel) and in vitro (right panel) nesfatin-1 and Nlp on glucose regulatory transcripts in goldfish adipose tissue. mRNA transcript abundance of *ins* (**A**,**B**), *preproglucagon* (**C**,**D**), *pcsk1* (**E**,**F**), *pcsk2* (**G**,**H**), *insra* (**I**,**J**), and *insrb* (**K**,**L**) following in vivo and in vitro nesfatin-1 and Nlp treatment in goldfish adipocytes. Data represent mRNA abundance (RT-qPCR) and are presented as mean + SEM (*n* = six fish/group, for both in vivo and in vitro groups). One-way ANOVA following Dunnett’s multiple comparisons test (in vivo study) or Tukey’s multiple comparisons test (in vitro study) were used for statistical analysis. Asterisks denote significant differences (* *p* < 0.05, ** *p* < 0.001, *** *p* < 0.0001) between control and treatment groups.

Both in vivo and in vitro treatment with nesfatin-1 and Nlp upregulated preproinsulin (*ins*) transcript abundance in goldfish adipocytes. In the in vivo treatment, a significant increase in *ins* mRNA expression was observed in both nesfatin-1- and Nlp-injected groups after one hour (Figure 1A) (*p* < 0.0001). Similarly, adipose tissue treated with nesfatin-1 and Nlp (10 nmol/L) showed a significant increase in *ins* transcript abundance after six hours (Figure 1B) (*p* < 0.0001). However, preproglucagon mRNA was significantly downregulated in adipocytes both in vivo (*p* < 0.001) and in vitro (*p* < 0.05) treatment groups (Figure 1C,D). The abundance of *pcsk1* and *pcsk2* mRNAs, formerly known as PC1/3 and PC2, which are involved in the bioprocessing of preprohormones (including preproinsulin) was significantly upregulated following nesfatin-1 and Nlp administration (*P <* 0.001) in goldfish adipocytes (Figure 1E,G). Similar to the results of the in vitro treatment, the mRNA abundance of *pcsk1* was significantly upregulated after six hours (10 nmol/L) for both nesfatin-1 and Nlp treatment (Figure 1F) (*p* < 0.001). *pcsk2* transcript abundance was significantly upregulated following nesfatin-1 (10 nmol/L, six-hour time point) treatment (*p* < 0.0001), whereas it was significantly downregulated following Nlp (1 nmol/L, one-hour time point) treatment (*p* < 0.05) (Figure 1H). IP injection of nesfatin-1 and Nlp significantly downregulated the transcript abundance of insulin receptors *insra* and *insrb* (Figure 1I,K). However, in vitro incubation with nesfatin-1 and Nlp significantly upregulated the transcript abundance of *insrb* at both one (1 nmol/L, *p* < 0.001) and six-hour time points (10 nmol/L, *p* < 0.0001) (Figure 1L), where no significant changes were observed for *insra* (Figure 1J).

### 3.2. Nesfatin-1 and Nlp Stimulated Glucose Transporter Transcript Abundance in Goldfish Adipose Tissue

**Figure 2:**
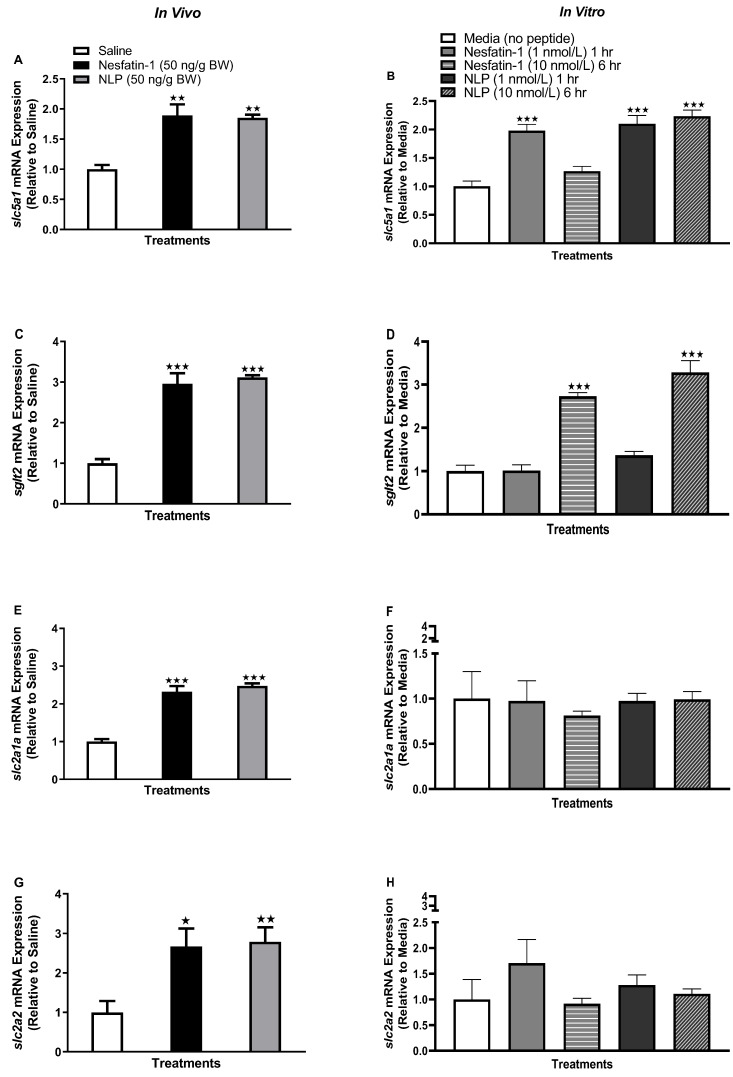
Glucose transporter transcript abundance in adipose tissue following in vivo (left panel) and in vitro (right panel) nesfatin-1 and Nlp treatment in goldfish. *slc5a1* (**A**,**B**), *sglt2* (**C**,**D**), *slc2a1a* (**E**,**F**), and *slc2a2* (**G**,**H**) transcript abundance profile in goldfish adipocyte following nesfatin-1 and Nlp treatment. mRNA abundance (RT-qPCR) data are presented as mean + SEM (*n* = six fish/group, for both in vivo and in vitro groups). One-way ANOVA following Dunnett’s multiple comparisons test (in vivo study) or Tukey’s multiple comparisons test (in vitro study) were used for statistical analysis. Asterisks denote significant differences (* *p* < 0.05, ** *p* < 0.001, *** *p* <0.0001) between control and treatment groups.

Following the IP administration of nesfatin-1 and Nlp, *slc5a1* (solute carrier family five member one, *sglt1*) mRNA abundance was significantly upregulated (Figure 2A) (*p* < 0.001). Similarly, in vitro incubation of goldfish adipocytes with nesfatin-1 and Nlp significantly upregulated *slc5a1* mRNA abundance at both the one-hour (1 nmol/L, *p* < 0.0001, except Nlp at one hour) and six-hour time points (10 nmol/L, *p* < 0.0001) (Figure 2B). Another glucose transporter, *sglt2* (sodium-glucose co-transporter 2), was also upregulated following nesfatin-1 and Nlp administration (Figure 2C) (*p* < 0.0001) and incubation (six hours, 10 nmol/L, *p* < 0.0001) (Figure 2D) in goldfish adipocytes. In vivo administration of nesfatin-1 and Nlp significantly upregulated *slc2a1a* (solute carrier family 2 member 1a, *glut1*) and *slc2a2* (solute carrier family 2 member 2, *glut2*) mRNA abundance in goldfish adipocytes (Figure 2E,G) (*p* < 0.0001 and *p* < 0.001, respectively). However, no significant effects were observed for *slc2a1a* or *slc2a2* following nesfatin-1 or Nlp in vitro treatment (Figure 2F,H).

### 3.3. Nesfatin-1 and Nlp Modulated an ghr-igf System mRNA Abundance in Goldfish Adipose Tissue

**Figure 3:**
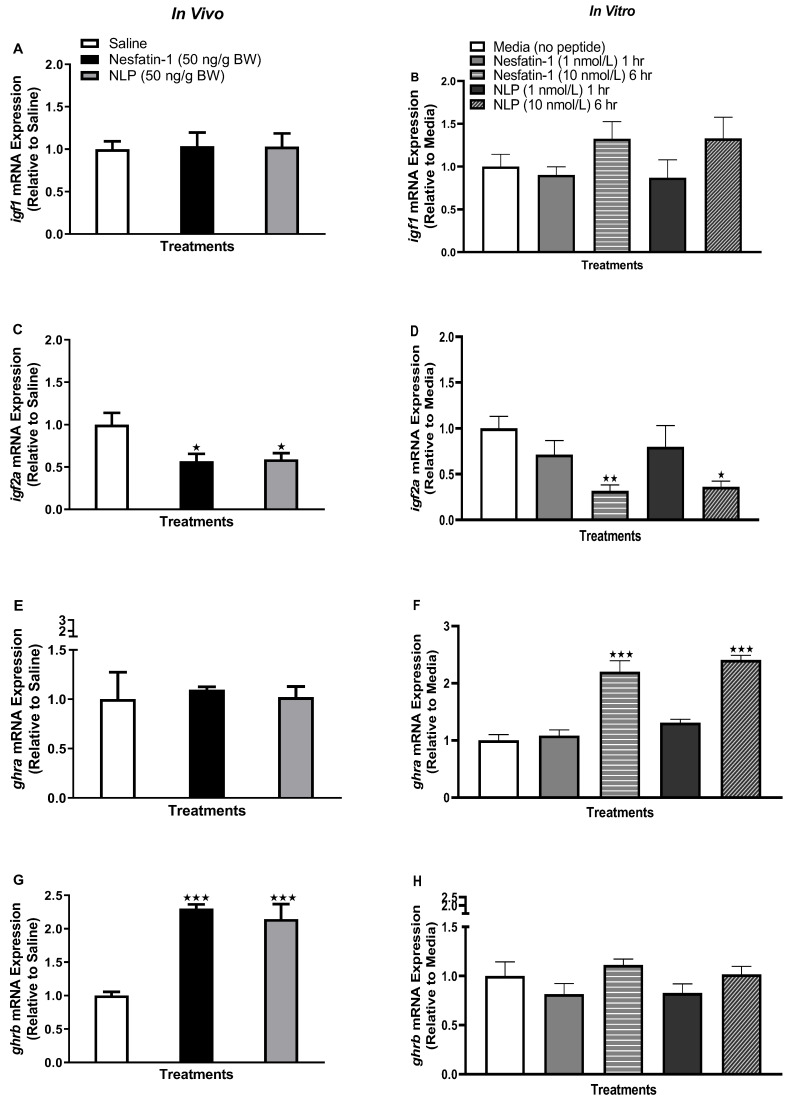
*Ghr-igf* transcript abundance following in vivo (left panel) and in vitro (right panel) nesfatin-1 and Nlp treatment in goldfish adipose tissue. Data represents mRNA abundance of *igf1* (**A**,**B**), *igf2a* (**C**,**D**), *ghra* (**E**,**F**), and *ghrb* (**G**,**H**) following nesfatin-1 or Nlp treatment. Data represent mRNA abundance (RT-qPCR) presented as mean + SEM (*n* = six fish/group, for both in vivo and in vitro groups). One-way ANOVA following Dunnett’s multiple comparisons test (in vivo study) or Tukey’s multiple comparisons test (in vitro study) were used for statistical analysis. Asterisks denote significant differences (* *p* < 0.05, ** *p* < 0.001, *** *p* < 0.0001) between control and treatment groups.

Following IP injection (one hour) and in vitro incubation of nesfatin-1 and Nlp (six hours, 10 nmol/L), a significant reduction in *igf2a* (insulin-like growth factor 2a) mRNA abundance was observed in goldfish adipocytes (Figure 3C,D) (*p* < 0.05). Another important Igf system gene, *igf1* (insulin-like growth factor 1) was unaffected following both (in vivo and in vitro) treatments (Figure 3A,B). The transcript abundance of *ghra* (growth hormone receptor a), an important receptor for Gh (growth hormone) signal transduction, was significantly upregulated following nesfatin-1 and Nlp incubation (at six hours, 10 nmol/L, *p* < 0.0001) (Figure 3F). Meanwhile, in vivo administration of nesfatin-1 or Nlp did not affect *ghra* mRNA abundance in goldfish adipocytes (Figure 3E). Growth hormone receptor b, or *ghrb*, another receptor for Gh, was significantly upregulated following IP administration of nesfatin-1 and Nlp (Figure 3G) (*p* < 0.0001). However, *ghrb* transcript abundance was unaffected by nesfatin-1 or Nlp treatment in vitro (Figure 3H).

### 3.4. Nesfatin-1 and Nlp Modulated ghr-igf System in Goldfish Liver

**Figure 4:**
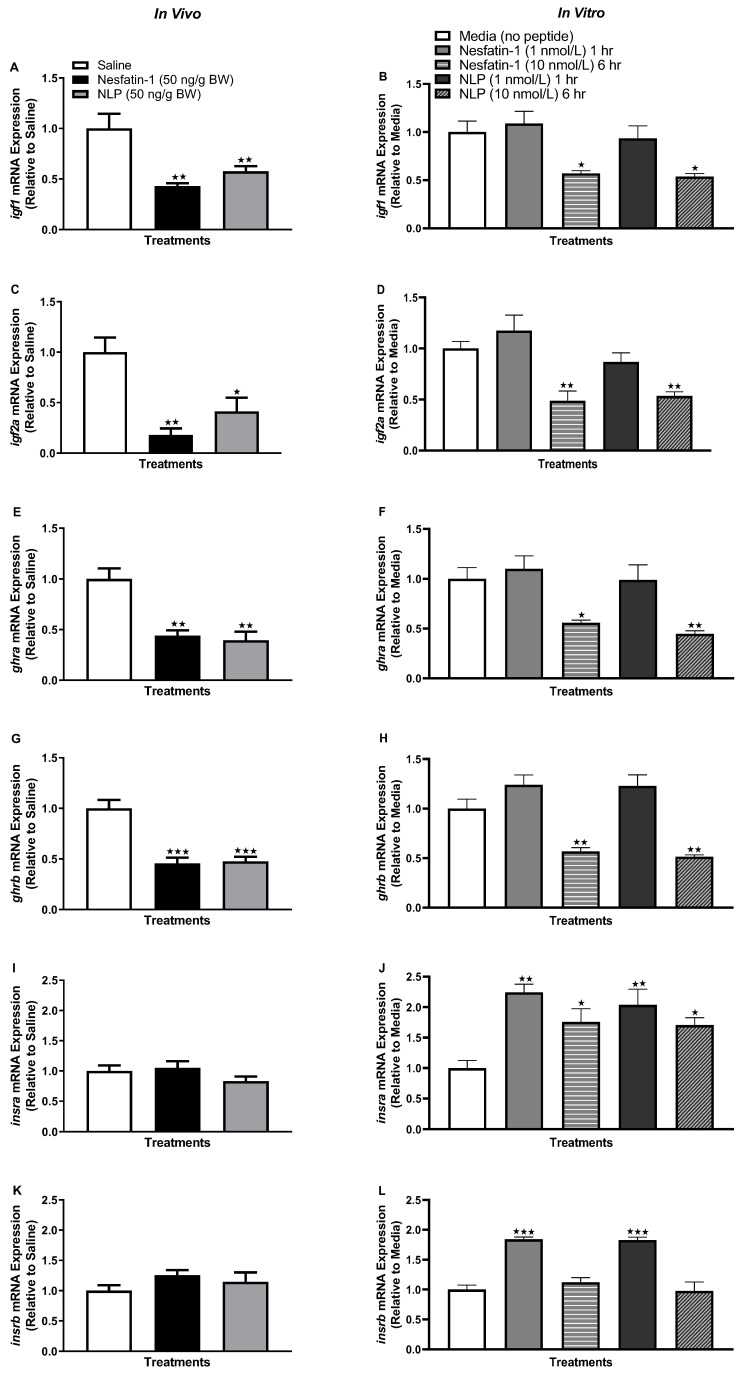
Insulin, growth hormone, and insulin-like growth factor receptor mRNA transcript following in vivo (left panel) and in vitro (right panel) nesfatin-1 and Nlp treatment in goldfish liver. Figure panel represents hepatic transcript profile of *igf1* (**A**,**B**), *igf2a* (**C**,**D**), *ghra* (**E**,**F**), *ghrb* (**G**,**H**) *insra* (**I**,**J**), and *insrb* (**K**,**L**) post-nesfatin-1 or Nlp treatment. RT-qPCR transcript data are presented as mean + SEM (*n* = six fish/group, for both in vivo and in vitro groups). One-way ANOVA following Dunnett’s multiple comparisons test (in vivo study) or Tukey’s multiple comparisons test (in vitro study) were used for statistical analysis. Asterisks denote significant differences (* *p* < 0.05, ** *p* < 0.001, *** *p* < 0.0001) between control and treatment groups.

IP injection of nesfatin-1 or Nlp significantly downregulated *igf1* mRNA in the liver of goldfish (*p =* 0.0009 for nesfatin-1 group and *p =* 0.0087 for Nlp group) (Figure 4A). Similarly, *igf2* mRNA was also downregulated following nesfatin-1 (*p =* 0.0005) and Nlp (*p =* 0.0070) administration (Figure 4C). In the in vitro treatment groups, nesfatin-1 and Nlp treatment significantly downregulated *igf1* (*p =* 0.0161) and *igf2* (*p =* 0.0033) mRNA expression at six-hours post-incubation (Figure 4B,D). The mRNA abundance of both *ghra* (*p =* 0.0005) and *ghrb* (*p* < 0.0001) were also downregulated following nesfatin-1 and Nlp administration in the liver of goldfish (Figure 4E,G). As observed for *igf1* and *igf2*, transcript abundance of both *ghra* (*p =* 0.0212 for nesfatin-1 and *p =* 0.0035 for Nlp treatment group compared to control) and *ghrb* (*p* < 0.001) was significantly downregulated at six-hours post-incubation (Figure 4F,H). In vitro incubation of either nesfatin-1 or Nlp upregulated *insra* transcript abundance at both one hour (*p =* 0.0014) and six hours (*p =* 0.0210) in goldfish liver tissue culture (Figure 4J). In addition, another insulin receptor transcript, *insrb,* was upregulated at the one-hour time point (*p* < 0.0001) following peptide incubation (Figure 4L). However, in vivo administration of nesfatin-1 and Nlp did not alter *insra* and *insrb* transcript abundance in the liver of goldfish (Figure 4I,K).

### 3.5. Nesfatin-1 and Nlp Differently Modulate the mRNA Abundance of Glucose Transporters in Goldfish Liver

**Figure 5:**
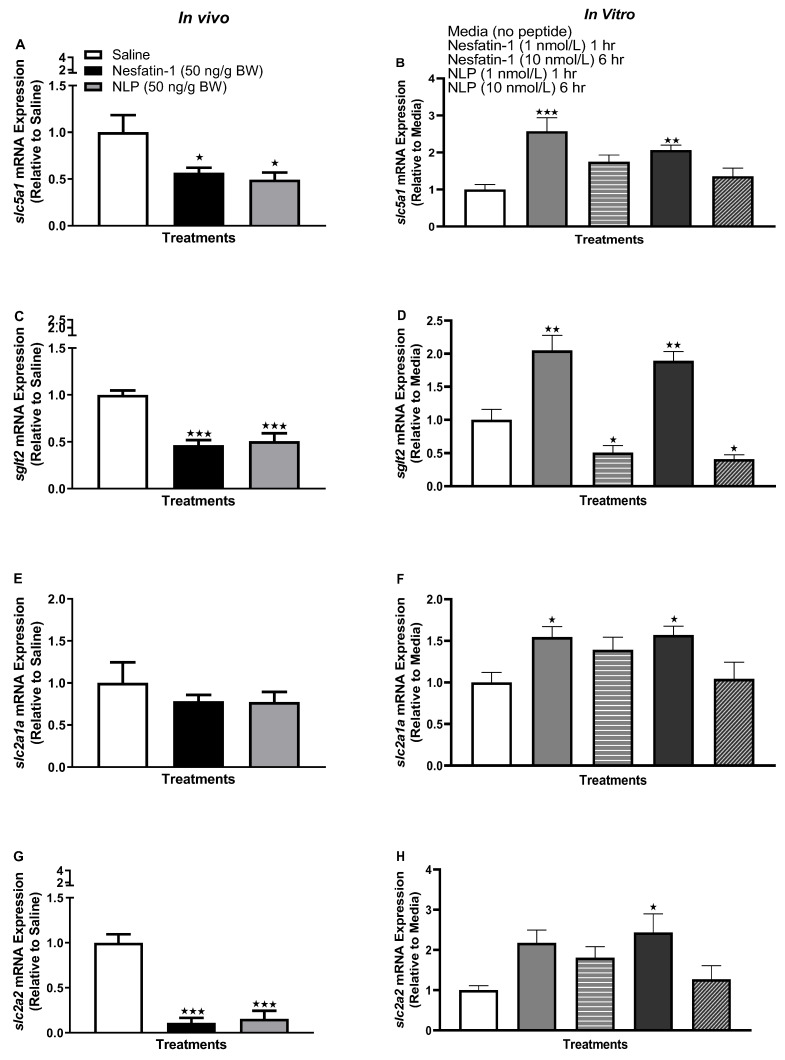
Transcript profile of glucose transporters following in vivo (left panel) and in vitro (right panel) nesfatin-1 and Nlp treatment in goldfish liver. RT-qPCR transcript data of *slc5a1* (**A**,**B**), *sglt2* (**C**,**D**), *slc2a1a* (**E**,**F**), and *slc2a2* (**G**,**H**) following nesfatin-1 and Nlp treatment in goldfish liver. Data presented as mean+ SEM (*n* = six fish/group, for both in vivo and in vitro groups). One-way ANOVA following Dunnett’s multiple comparisons test (in vivo study) or Tukey’s multiple comparisons test (in vitro study) were used for statistical analysis. Asterisks denote significant differences (* *p* < 0.05, ** *p* < 0.001, *** *p* < 0.0001) between control and treatment groups.

IP injection of nesfatin-1 and Nlp downregulated *slc5a1* mRNA in the liver of goldfish (*p =* 0.0164) (Figure 5A). However, in vitro treatment of nesfatin-1 (*p =* 0.0002) and Nlp (*p =* 0.0091) upregulated *slc5a1* mRNA in the liver culture at one-hour post-incubation (Figure 5B). *Sglt2* mRNA was also downregulated following nesfatin-1 and Nlp injection (*p* < 0.0001) in the liver of goldfish (Figure 5C). At one hour (1 nmol/L), incubation in nesfatin-1 or Nlp significantly upregulated (*p* < 0.0001) the abundance of *sglt2* (Figure 5D). However, at six hours (10 nmol/L), *sglt2* was significantly downregulated (*p =* 0.0336) following the peptide treatment compared to the control (Figure 5D). *Slc2a1a*, another glucose transporter, was upregulated at the one-hour time point following nesfatin-1 and Nlp incubation (*p =* 0.0334) (Figure 5F). However, no significant changes were observed in the transcript profile of *slc2a1a* following IP injection of either nesfatin-1 or Nlp (Figure 5E). Nesfatin-1 and Nlp downregulated *slc2a2* (*p* < 0.0001) at one-hour (Figure 5G) post-injection. Meanwhile, the Nlp in vitro treatment at the one-hour time point caused a significant increase (*p =* 0.0145) in *slc2a2* transcript abundance compared to controls (Figure 5H).

### 3.6. IP Injection, Not In Vitro Treatment with Nesfatin-1 or Nlp, Upregulated the mRNA Abundance of ghr-igf System in the Muscle of Goldfish

**Figure 6:**
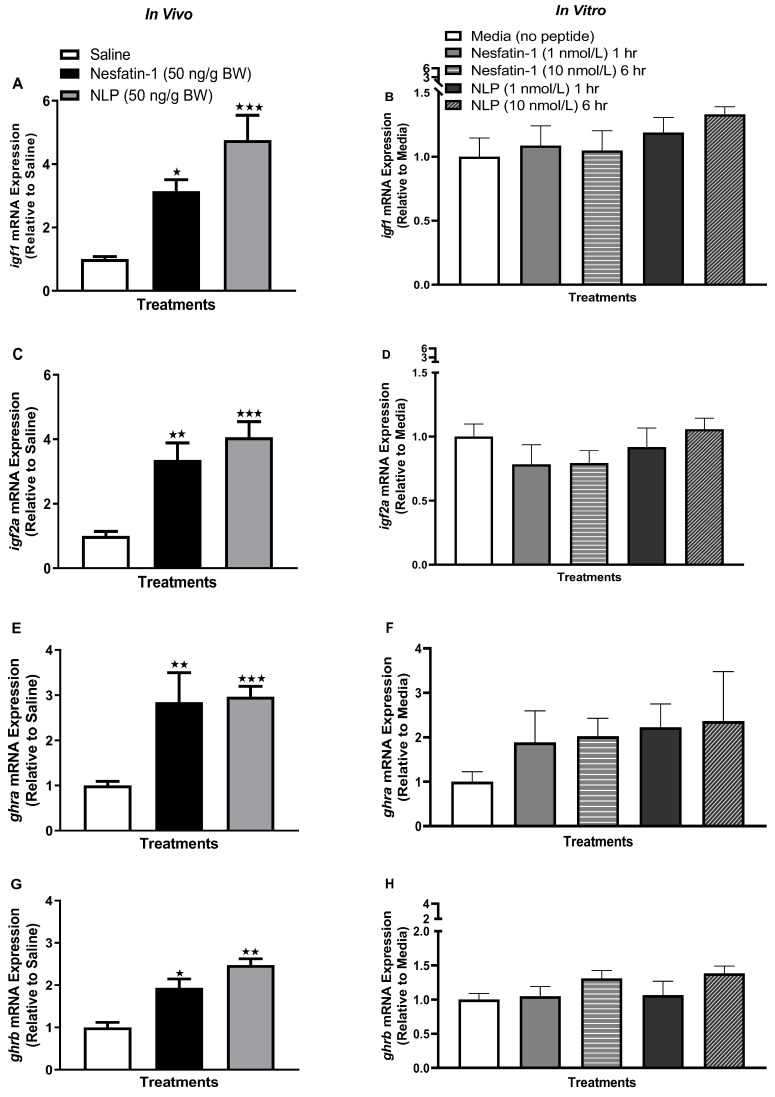
Representative graphs showing the transcript profile of *ghr-igf* mRNAs in goldfish skeletal muscle following in vivo (left panel) and in vitro (right panel) nesfatin-1 and Nlp treatment. Transcript profile of *igf1* (**A**,**B**), *igf2a* (**C**,**D**), *ghra* (**E**,**F**), and *ghrb* (**G**,**H**) post-nesfatin-1 and Nlp treatment. Data were obtained by RT-qPCR and are presented as mean+ SEM (n = six fish/group, for both in vivo and in vitro groups). One-way ANOVA following Dunnett’s multiple comparisons test (in vivo study) or Tukey’s multiple comparisons test (in vitro study) were used for statistical analysis. Asterisks denote significant differences (* *p* < 0.05, ** *p* < 0.001, *** *p* < 0.0001) between control and treatment groups.

IP injection (in vivo) of nesfatin-1 and Nlp upregulated the transcript abundance of *igf1* (*p =* 0.0158 and 0.0002 for nesfatin-1 and Nlp), i*gf2a* (*p =* 0.0025 and 0.0003), *ghra* (*p =* 0.0023 and *p* < 0.0001), and *ghrb* (*p =* 0.0108 and 0.0070) in the muscle of goldfish at the one-hour time point (Figure 6A,C,E,G). However, no significant changes in the abundance of *igf1*, i*gf2a*, *ghra,* and *ghrb* mRNAs in muscle were observed following nesfatin-1 and Nlp treatment in vitro (Figure 6B,D,F,H).

### 3.7. Nesfatin-1 and Nlp Upregulated the Transcript Abundance of Glucose Transporters in Goldfish Muscle

**Figure 7:**
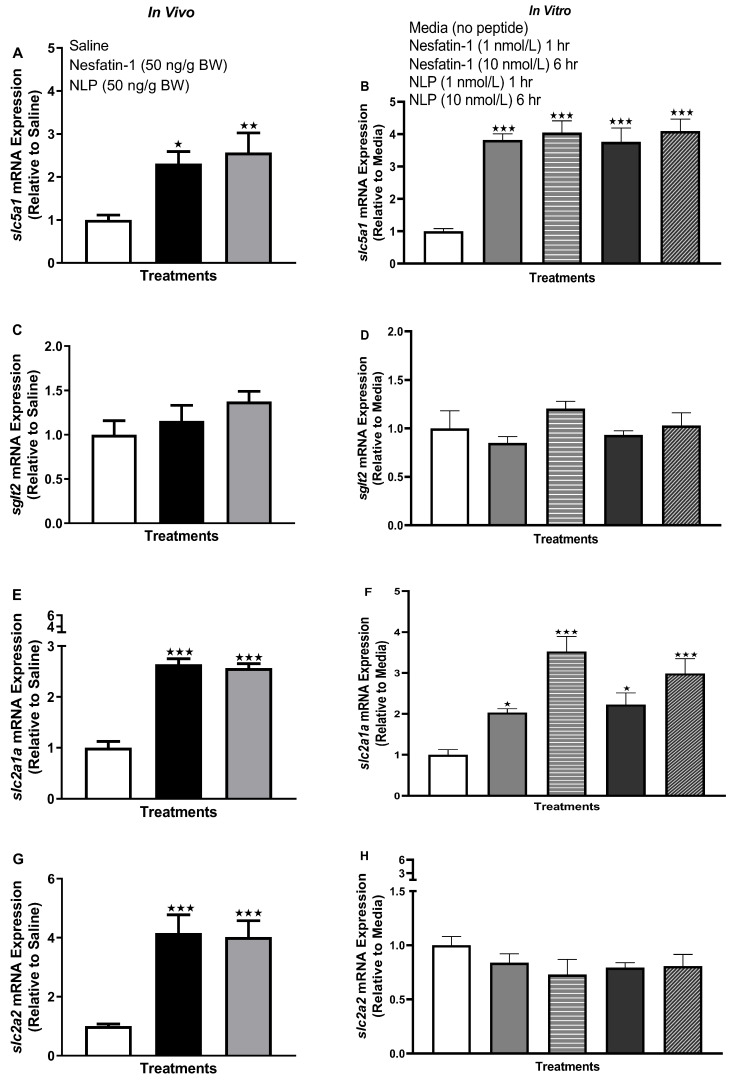
Skeletal muscle glucose transporter transcript abundance following in vivo (left panel) and in vitro (right panel) nesfatin-1 and Nlp treatment. RT-qPCR transcript abundance profile of *slc5a1* (**A**,**B**), *sglt2* (**C**,**D**), *slc2a1a* (**E**,**F**), and *slc2a2* (**G**,**H**) in goldfish skeletal muscle following nesfatin-1 and Nlp treatment. Data are presented as mean + SEM (*n* = six fish/group, for both in vivo and in vitro groups). One-way ANOVA following Dunnett’s multiple comparisons test (in vivo study) or Tukey’s multiple comparisons test (in vitro study) were used for statistical analysis. Asterisks denote significant differences (* *p* < 0.05, ** *p* < 0.001, *** *p* < 0.0001) between control and treatment groups.

Following IP injection of nesfatin-1 or Nlp, *slc5a1* mRNA was significantly upregulated at the one-hour time point in goldfish muscle (*p =* 0.0193 and 0.0060 for nesfatin-1 and Nlp) (Figure 7A). Similar to in vivo administration, in vitro incubation with nesfatin-1 and Nlp significantly upregulated the transcript abundance of *slc5a1* in goldfish muscle (*p* < 0.0001) at both the one and six-hour time points (Figure 7B). However, *sglt2* was unaffected by either nesfatin-1 or Nlp in both in vivo and in vitro treatment groups (Figure 7C,D). Both *slc2a1a* and *slc2a2* were also significantly upregulated following the IP administration of nesfatin-1 and Nlp in goldfish muscle (*p* < 0.0001) (Figure 7E, G). Similarly, *slc2a1a* mRNA abundance was significantly upregulated after both one hour (*p =* 0.0142) and six hours (*p* < 0.0001) in goldfish muscle following nesfatin-1 and Nlp incubation (Figure 7F). However, no significant changes in *slc2a2* transcript abundance were found after nesfatin-1 and Nlp incubation in goldfish muscle in vitro (Figure 7H).

### 3.8. Nesfatin-1 and Nlp Upregulated the Insulin Receptor Transcript Abundance in Goldfish Muscle

**Figure 8:**
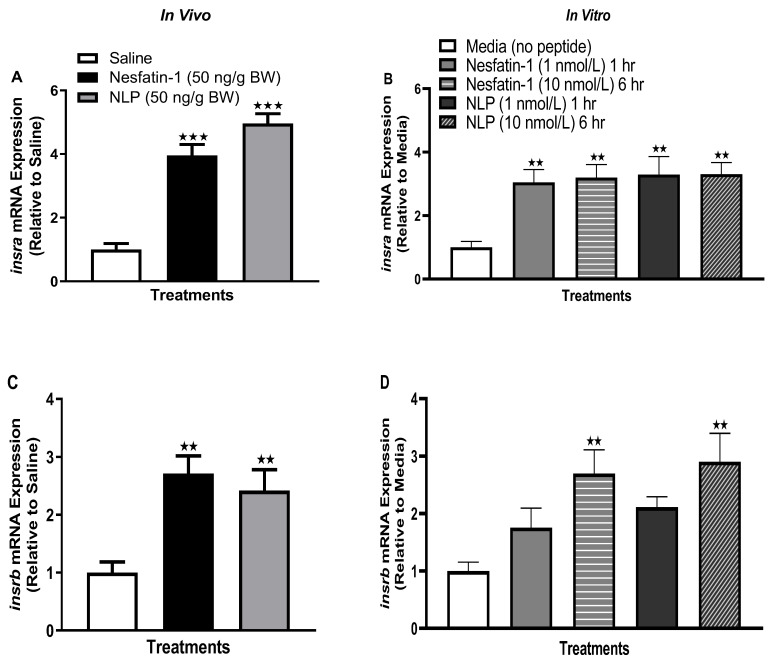
Insulin receptor mRNA abundance following in vivo (left panel) and in vitro (right panel) nesfatin-1 and Nlp treatment. *Insra* (**A**,**B**) and *insrb* (**C**,**D**) transcript abundance in goldfish skeletal muscle following nesfatin-1 and Nlp treatment. Graphs represent RT-qPCR mRNA abundance presented as mean + SEM (*n* = six fish/group, for both in vivo and in vitro groups). One-way ANOVA following Dunnett’s multiple comparisons test (in vivo study) or Tukey’s multiple comparisons test (in vitro study) were used for statistical analysis. Asterisks denote significant differences (** *p* < 0.001, *** *p* < 0.0001) between control and treatment groups.

IP administration of nesfatin-1 or Nlp caused a significant increase in the transcript abundance of both *insra* (*p* < 0.0001) and *insrb* (*p =* 0.0021) in the muscle of goldfish (Figure 8A,C). Similarly, in vitro incubation of goldfish muscle with nesfatin-1 or Nlp upregulated both *insra* (at both one- and six-hour time points, *p* < 0.001) and *insrb* (only at the six-hour time point, *p* < 0.001) transcripts (Figure 8B,D).

## 4. Discussion

Nesfatin-1 and Nlp are key players in the regulation of growth [3,15], feeding [1,2,7,16], reproduction [4,5,8], heart function [6,17], and hormone secretion [3,4,5,8,9,10,15,18] in fish and mammals. Overall, nesfatin-1 and Nlp are multifunctional peptides with tissue-specific, hormone-like actions in fish and mammals. In the present study, we used goldfish and both in vivo and in vitro approaches to further elucidate the role of nesfatin-1 and Nlp in three metabolic tissues: liver, muscle, and the adipose tissue. The role of nesfatin-1 in the regulation of pancreatic function and insulin secretion was studied in detail in mammals [16,18,19]. Nesfatin-1 as a biomarker for diabetes mellitus and related disorders is being considered [20,21,22,23]. Recently, it was reported that the mesenteric adipocytes from goldfish are an alternative source of insulin and have pancreatic β-cell-like functions [10]. A distinct endocrine pancreatic tissue is lacking in many fish species [24,25] and there is limited insulin responsiveness to a glucose load in fish [7,26]. However, all major energy sources, including glucose [27,28], fatty acid [29,30,31], and amino acids [32,33], are known to influence insulin functions in fish. We employed both in vivo and in vitro administration of synthetic nesfatin-1 and Nlp and found that both treatments significantly upregulate preproinsulin (*ins*) mRNA abundance in goldfish adipocytes, a newly identified source of insulin in goldfish. The effects found were similar to nesfatin-1 and Nlp actions reported previously in rodents and in murine pancreatic beta-like cells (MIN6) [18,34]. This suggests that the insulinotropic action of nesfatin-1 and Nlp is likely conserved in fish and mammals.

In addition to the *ins* transcripts, nesfatin-1 and Nlp upregulated *pcsk1* and *pcsk2* mRNAs. This suggests possible positive roles for nesfatin-1 and Nlp on proinsulin processing. The transcript abundance of *preproglucagon* mRNA was significantly downregulated following nesfatin-1 and Nlp treatment (both in vivo and in vitro). IP administration of nesfatin-1 upregulated the abundance of *preproglucagon* in the pancreas of mice [35] and in intestinal (STC-1) cells [36] in vitro. However, the decrease in *preproglucagon* in the adipose tissue observed here suggests glucagon suppression as another mode of action of nesfatin-1 and Nlp to regulate glucose homeostasis in fish. In addition, an increase in glucose transporter mRNAs was also found. This suggests that both nesfatin-1 and Nlp potentially increase the clearance of glucose from circulation by activating glucose uptake machinery in adipose tissue through insulin and glucose transporters. These results agree with the outcomes of studies conducted in mice, where a continuous infusion of nesfatin-1 upregulated both insulin and glucose transporters (Glut4 or slc*2a4*) [37] in the muscle and adipose tissue. The modes of action of nesfatin-1 on glucose homeostasis appear conserved across species.

A role for nesfatin-1 and Nlp in the regulation of the Gh-Igf system was found and both peptides downregulated the Igf system (*igf2a*), while the growth hormone receptor (*ghra* and *ghrb*) was found upregulated. The transcript abundance data from the adipose tissue suggest that both nesfatin-1 and Nlp cause an overall increase in glucose uptake and energy conservation in the form of lipid storage and a decrease in growth. It is to be noted that this study was conducted in 24 h-fasted fish, when metabolic hormones are probably driving the animal towards an energy conservation mode. It was reported that nesfatin-1 negatively influences the Gh-Igf axis by downregulating Gh synthesis and release, as well as by decreasing hepatic *igf1* and *igf2* mRNA [3]. We further tested this in goldfish liver using in vivo and in vitro methods and found that both nesfatin-1- and Nlp-treated groups show a significant decrease in Gh receptors (*ghra* and *ghrb*) as well as Igf (*igf1* and *igf2a*) mRNA abundance. This further confirms that both nesfatin-1 and Nlp are negative regulators of the Gh-Igf axis in goldfish. In addition, insulin receptors (*insra* and *insrb*) (in vitro) as well as glucose transporter transcript (*slc5a1*, *sglt2*, *slc2a1a,* and *slc2a2*) abundance (in vitro) were significantly upregulated (except *sglt2* at the six-hour time point) following nesfatin-1 and Nlp treatment. This suggests a direct action (upregulation) of nesfatin-1 and Nlp on glucose transporter transcript abundance. However, this effect was absent in the in vivo group and an overall decrease in glucose transporter transcript abundance was observed. Our results on the transcript abundance of glucose transporters and insulin signaling suggest a direct stimulatory effect in vitro, but an indirect in vivo inhibitory role for nesfain-1 and Nlp on the hepatic glucose metabolism.

The third tissue studied, skeletal muscle, is the major insulin-responsive and glucose-disposing organ in mammals [38], and to some extent in fish. All glucose transporters tested (except *sglt2* and *slc2a2* in vitro) and insulin receptors were significantly upregulated following both in vivo and in vitro nesfatin-1 or Nlp treatments, suggesting that both nesfatin-1 and Nlp promote glucose uptake in the muscle of goldfish. This is similar to what we observed in adipose tissue, where nesfatin-1 and Nlp promote glucose transporter transcript abundance. It was reported that peripheral infusion of nesfatin-1 significantly increases both skeletal muscle and adipose tissue Glut4 translocation in mice [37]. This suggests that the glucose transporter regulatory mechanism of nesfatin-1 is conserved across species (for both adipose tissue and muscle). In addition, this study confirms that Nlp also causes the same effect reported for nesfatin-1 previously, further supporting that Nlp is indeed a “nesfatin-1-like” peptide. Unlike the transcript abundance response that we observed in the liver, following nesfatin-1 and Nlp administration (only in in vivo group), all Gh-Igf transcripts tested were significantly upregulated after one hour. This was not observed in nesfatin-1 and Nlp incubated groups (in vitro), where no significant changes in Gh-Igf transcripts were observed at both time points tested (one and six hours).

## 5. Conclusions

Overall, the results of this research reveal additional tissue-specific actions of nesfatin-1 and Nlp in goldfish. The upregulation of skeletal muscle Gh-Igf (in vivo), as well as the overall increase in glucose transporter (both in vivo and in vitro) transcript abundance data, further confirms the tissue-specific and conserved regulatory role of both nesfatin-1 and Nlp in goldfish. A limitation of this research is that it lacks any protein or hormone data. However, here we combined and compared the transcript data from hormone targets (preproinsulin and *preproglucagon*), their processing enzymes (*pcsk1* and *pcsk2*), downstream targets (receptors, *insra*, *insrb, ghra* and *ghrb*), as well as carrier molecules (glucose transporters). This appears to be the first comprehensive set of data on the roles of nesfatin-1 and Nlp on insulin and gluco- and growth-regulatory factors in three metabolic tissues of any species where these peptides were studied before. It provides new insights on Nlp effects in fish. Our results suggest that, largely, the roles of both nesfatin-1 and Nlp on the aspects studied here are conserved in goldfish, a well characterized model in neuroendocrinology. Future research should aim to determine how these changes in metabolic and growth regulators translate into growth and bodyweight in goldfish following chronic administration of nesfatin-1 or Nlp.

## Figures and Tables

**Table 1:** Primer details, including sequence information, GenBank accession numbers, and annealing temperatures employed in qPCR.

Gene	Accession No.	Primer Sequence (5′-3′)		Annealing Temperature (°C)
		Forward	Reverse	
18S rRNA	MG830470.1	GGATGCCCTTAACTGGGTGT	CTAGCGGCGCAATACGAATG	60
β-actin	LC382464.1	CAGGGAGTGATGGTTGGCA	AACACGCAGCTCGTTGTAGA	60
*ghra*	XM_026270357.1	CGCCAATGATTCCCAGACG	ATGGGCATGGTTGGGATTACA	60
*ghrb*	KT985189.1	TCCACCAGTGATTCCCAGACG	GGTAGGCATTGCTGGGAGGT	60
*igf1*	GU583648.1	GGGGGCAGAAACTATCGCAT	GCACGTCCCTGCAAAAATTCA	60
*igf2a*	FJ410929.1	CGTGCCGAAAAACTGTGGAA	CTCCGCACACGAACTGAAGA	57
*slc2a1a*	XM_026265785.1	TGGCCTTCTTTGAGATTGGACC	ACTTTGAAGTAGGTGAAGACGAAGAA	58
*slc2a2*	XM_026206696.1	TGTGCTGTGGCCATGAC	CCAGGTCCGATCTCAAAGAA	58
*slc5a1*	XM_026274578.1	GATCGTGACCATGCCAGAG	TTTAGTCCCAGAGCCTGGTT	58
*sglt 2*	XM_026264357.1	GCACCTTGTTCACCATGGACAT	ACCACTCTGGGCTGCCTG	58
*preproinsulin*	LC387783.1	ATCACGCCGAGCTGATAAGG	TGGTGAAGTCATTGGCGGTT	57
*preproglucagon*	U65528.1	GCCTGGCTAAAATCCGGACA	CGTGATGAAGCAGTCAGCAG	60
*pcsk1*	XM_026196557.1	ATCGTGGTCATCCAGGTCAT	CGCACTTCTTTTGGTCCTGC	58
*pcsk2*	NM_001142266.1	GTCAGAAGCGAGGCTACAGA	CCATCATCCATAATGGCGATGGT	58
*insra*	AF218355.1	GCCCACCTTGAAGGAGATGA	TTCCGAAGTCGCCAATCTTCA	60
*insrb*	AF321225.1	GAGCTGCACCATTGCGTATC	CCACACGTAGGTCTTGCACA	60

## Data Availability

The data presented in this study are available upon reasonable request from the corresponding author.
